# High levels of polyandry, but limited evidence for multiple paternity, in wild populations of the western rock lobster (*Panulirus cygnus*)

**DOI:** 10.1002/ece3.3985

**Published:** 2018-04-10

**Authors:** Jacqueline Loo, Winn Jason Kennington, Simon de Lestang, Jason How, Jonathan P. Evans

**Affiliations:** ^1^ Centre for Evolutionary Biology School of Biological Sciences The University of Western Australia Crawley WA Australia; ^2^ Western Australian Fisheries and Marine Research Laboratories North Beach WA Australia

**Keywords:** cryptic female choice, postcopulatory sexual selection, sexual selection, spawning, sperm competition

## Abstract

Polyandry, where multiple mating by females results in the temporal and spatial overlap of ejaculates from two or more males, is taxonomically widespread and occurs in varying frequencies within and among species. In decapods (crabs, lobsters, crayfish, and prawns), rates of polyandry are likely to be variable, but the extent to which patterns of multiple paternity reflect multiple mating, and thus are shaped by postmating processes that bias fertilization toward one or a subset of mated males, is unclear. Here, we use microsatellite markers to examine the frequency of multiple mating (the presence of spermatophores from two or more males) and patterns of paternity in wild populations of western rock lobster (*Panulirus cygnus*). Our data confirm that >45% of females had attached spermatophores arising from at least two males (i.e., confirming polyandry), but we found very limited evidence for multiple paternity; among 24 clutches sampled in this study, only two arose from fertilizations by two or more males. Single inferred paternal genotypes accounted for all remaining progeny genotypes in each clutch, including several instances when the mother had been shown to mate with two or more males. These findings highlight the need for further work to understand whether polyandry is adaptive and to uncover the mechanisms underlying postmating paternity biases in this system.

## 1 INTRODUCTION

Polyandry, where females mate with two or more males, is taxonomically widespread (Birkhead & Møller, 1998) despite potential costs to females, such as wasted time and energy (Watson, Arnqvist, & Stallmann, 1998), increased predation risk (Rowe, 1994), exposure to disease (Thrall, Antonovics, & Dobson, 2000), and risk of injuries (Crudgington & Siva‐Jothy, 2000). The maintenance of polyandry has been attributed to material (direct) and genetic (indirect) benefits for females. Direct benefits include fertility assurance, provision of resources and parental care for the offspring (Sheldon 1994). Indirect benefits include genetic incompatibility avoidance, increased genetic diversity of offspring, and the enhanced survival and reproductive success of offspring (Jennions & Petrie, 2000; Neff & Pitcher, 2005; Tregenza & Wedell, 2000). Polyandry may also occur in the absence of benefits to females in order to avoid the costs of male harassment (“convenience polyandry”; Thornhill & Alcock, 1983).

A common assumption in the literature in sexual selection is that polyandry will inevitably lead to multiple paternity. Indeed, multiple paternity, as estimated by assigning parentage of offspring from putatively multiply mated females, is commonly used to estimate the level of polyandry in natural populations (Taylor, Price, & Wedell, 2014). Yet, polyandry does not always translate into multiple paternity, as a number of postmating processes can ultimately determine which males are successful at fertilizing a female's eggs. For example, polyandry provides the scope for postmating episodes of sexual selection in the form of sperm competition (Parker, 1970) and/or cryptic choice (Eberhard, 1996; Thornhill, 1983), which have the potential to affect fertilization outcomes (Pizzari & Wedell, 2013). Sperm competition is the competition between sperm of different males to fertilize a female's eggs (Parker, 1970), whereas cryptic choice occurs when females influence the outcome of sperm competition (Eberhard, 1996; Thornhill, 1983). Sperm competition and cryptic female choice play critical roles in postmating sexual selection and have important consequences at both population and individual levels (Birkhead & Pizzari, 2002).

The mating systems of decapod crustaceans are highly diverse and complex (Duffy & Thiel, 2007; Martin, Crandall, & Felder, 2016). In many species, reproduction is synchronized with the molt cycle, with females being receptive only for a limited time after molting (Duffy & Thiel, 2007). Females approaching their reproductive molt are often guarded by males for one to several days before copulation (Duffy & Thiel, 2007; Subramoniam, 2013). Precopulatory male guarding is considered an evolutionary response to time‐limited opportunity for fertilization and to the need to protect recently molted females (Duffy & Thiel, 2007). In species with external fertilization (e.g., lobsters), males attach their spermatophores on the sternal plates of the female's cephalothorax during mating (Phillips, Cobb, & George, 2012). After mating, postmating guarding by the male occurs in some species, presumably to reduce the risk that females will mate with other males (Duffy & Thiel, 2007).

Parentage studies have revealed that polyandry is widespread in decapods and that there is substantial variation in the extent of multiple paternity within and among species, ranging from zero in the European lobster, *Homarus gammarus* (Ellis et al., 2015) to 100% in the squat lobster, *Munida sarsi* (Bailie, Hynes, & Prodohl, 2011). For the most part, however, parentage studies on decapods have been conducted on crabs (Baggio et al., 2011; Jensen & Bentzen, 2012; Jossart et al., 2014; Koga, Henmi, & Murai, 1993; McKeown & Shaw, 2008; Pardo, Riveros, Fuentes, Rojas‐Hernandez, & Veliz, 2016; Reaney, Maurer, Backwell, & Linde, 2012; Sainte‐Marie, Gosselin, Sevigny, & Urbani, 2008). In some cases, where multiple paternity has been detected, considerable skews in fertilization success toward a single male have been reported (Bailie et al., 2011, 2014; Gosselin, Sainte‐Marie, & Bernatchez, 2005; Plough, Moran, & Marko, 2014). Such skew may result from a range of postmating processes, including cryptic female choice (Thiel & Hinojosa, 2003) and sperm competition (Diesel, 1990; Sévigny & Sainte‐Marie, 1996; Urbani, Sainte‐Marie, Sévigny, Zadworny, & Kuhnlein, 1998). For example, in crabs of the infraorder Brachyura, in which spatial segregation of multiple paternal ejaculates has been reported, the anatomical structure of the spermathecae increases the probability of fertilization for the last male, that is, last‐male precedence (Jensen & Bentzen, 2012). Furthermore, in freshwater crayfish, males bias paternity in their favor by depositing sperm plugs (Holdich, Reeve, Holdich, & Lowery, 1988) diluting sperm (Rubolini et al., 2007) and removing or displacing sperm from previous males (Villanelli & Gherardi, 1998). By comparison, we know little about the mating system of lobsters (Ellis et al., 2015; Gosselin et al., 2005; Melville‐Smith, de Lestang, Beale, Groth, & Thompson, 2009; Streiff, Mira, Castro, & Cancela, 2004), especially regarding female mating strategies and the prevalence of polyandry and multiple paternity in natural populations.

**Figure 1:**
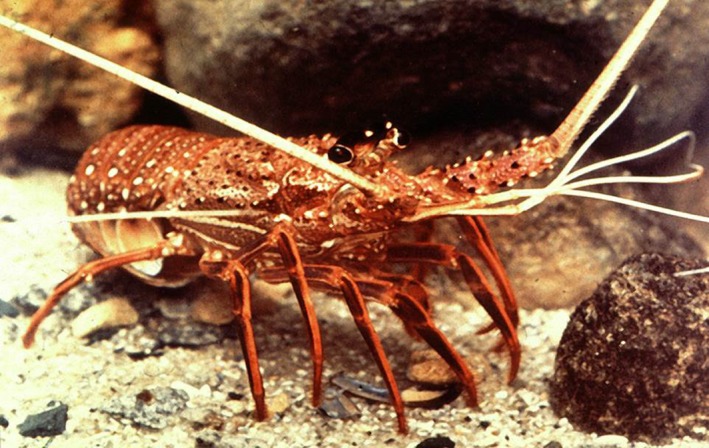
The western rock lobster (*Panulirus cygnus*). Photograph courtesy of the Western Australian Department of Primary Industry and Regional Development

The western rock lobster (*Panulirus cygnus*) is endemic to the Indo‐West Pacific Ocean region (see Figure 1). It is found in temperate to subtropical waters along the Western Australian coastline, ranging from Exmouth (21°55′ 59″S, 114°7′41″E) in the north to Albany in the south (35°1′22″S, 117°52′53″E) (Phillips, 2013). The reproductive behavior and life cycle of *P. cygnus* is described in detail elsewhere (Chittleborough, 1976; Phillips, 2013). Briefly, the spawning season commences in early spring, when males attach their spermatophores (sperm packets, typically termed “tar spots”) to the sternums of receptive females. Fertilization takes place when females extrude eggs and scratch the spermatophoric mass to release motile sperm. Remnants of the attached tar spots remain until they are either covered by a second mating or removed during molting. The life cycle of *P. cygnus* includes a long (~9–11 months) oceanic larval phase, during which planktonic phyllosoma larvae disperse as far as 1,500 km offshore. Helped by favorable winds and currents, the larvae subsequently return to the continental shelf where the final‐stage larvae metamorphose into the puerulus (postlarvae) that swim toward the shore and settle in shallow reefs. The settled pueruli develop into juveniles and subsequently adults in 5–6 years.

Here we provide new insights into the mating systems and reproductive behavior of *P. cygnus*, which until now has been limited mainly to observations conducted under laboratory controlled conditions (Chittleborough, 1974, 1976). Our recent work on wild populations of *P. cygnus* (J. Loo et al. unpubl. data) found evidence of high levels of polyandry in natural populations, with up to 52% of mated females at some locations carrying spermatophores from two or more males. However, this previous study did not genotype fertilized eggs and therefore was unable to confirm whether multiple mating translated into multiple paternity. In this study, we use microsatellite markers to examine patterns of paternity in two wild populations of *P. cygnus*. By focusing on both singly and multiply mated females (i.e., females carrying spermatophores from one male or two or more males, respectively), we are able to test whether multiple mating leads to multiple paternity. In this way, our study combines data on multiple mating *and* offspring paternity to provide insights into the likely importance of postmating sexual selection in this system.

## 2 MATERIALS AND METHODS

### 2.1 Sampling

**Figure 2:**
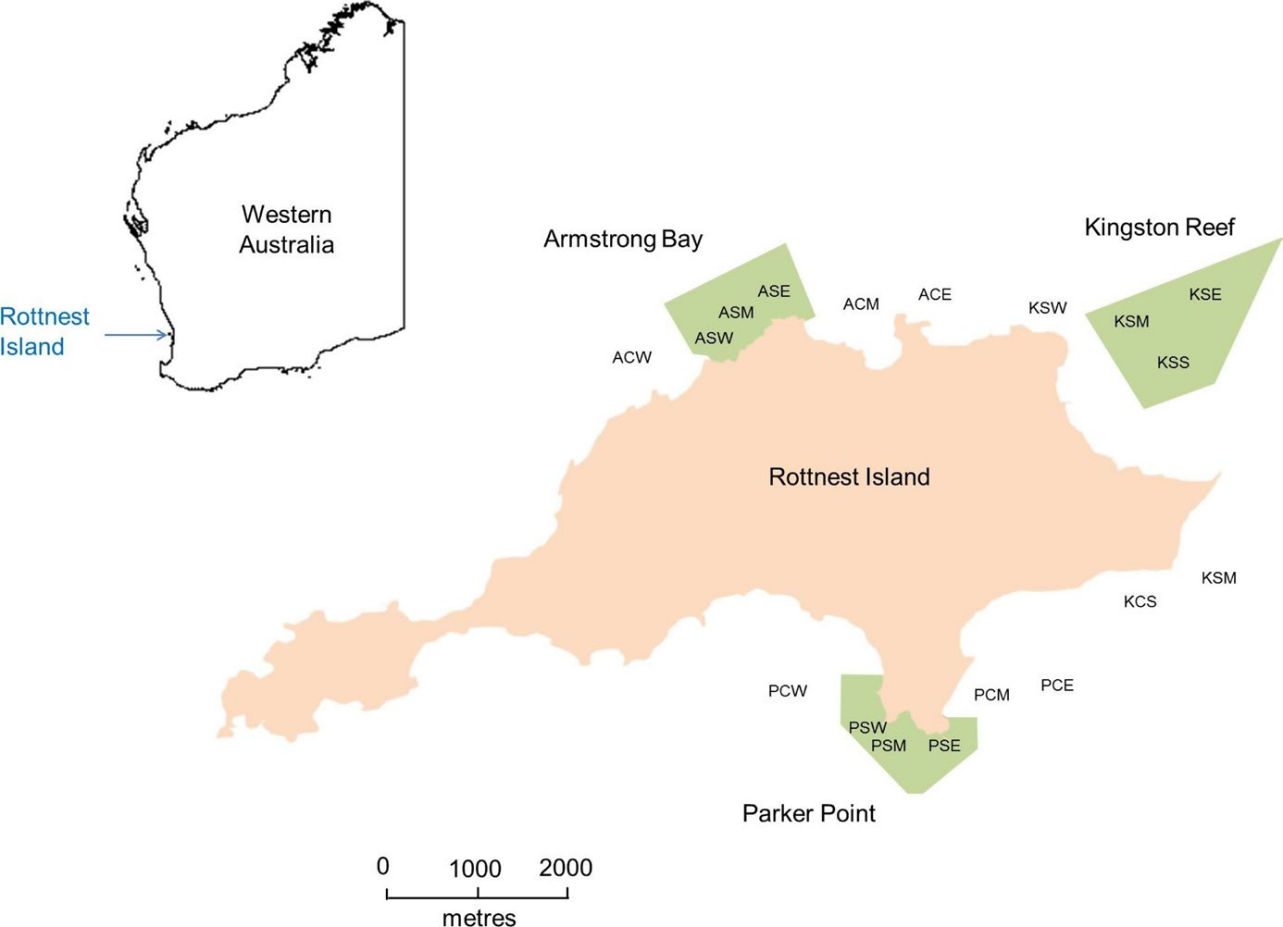
Map showing the sampling sites at Rottnest Island. The areas shaded green represent marine protection zones

The study was conducted in Rottnest Island, located 18 km west of Fremantle, in south‐west Western Australia (32°00′S, 115°30′E). Sampling was conducted over 16 days in February 2015 by the West Australian Department of Primary Industries and Regional Development; Fisheries Division as part of their regular monitoring program. Lobsters were sampled at two locations (Figure 2), using dive and pot‐based survey techniques (Bellchambers et al., 2009).

For each lobster captured during these collections, the sex and carapace length (CL, measured to the nearest 0.1 mm using a dial caliper) were recorded. Tissue samples from pleopods were collected from all males with a CL >64.5 mm (the minimum CL of a mature male reported at Lancelin; Melville‐Smith & de Lestang, 2006) and from females carrying spermatophores and/or eggs. A small piece of spermatophore and a cluster of eggs were removed from females. All tissue samples were preserved in 100% ethanol.

### 2.2 DNA extraction

Total genomic DNA was extracted from spermatophores using DNeasy Blood and Tissue kit (QIAGEN) following the manufacturer's protocol. Total genomic DNA was extracted from pleopods and individual eggs using proteinase K digestion followed by a DNA extraction method using DNA binding plates (Pall Corporation), as described in Ivanova, Dewaard, and Hebert (2006). The concentration and quality of the DNA of each sample was quantified using a NanoDrop ND‐1000 spectrophotometer.

Samples were genotyped at seven microsatellite loci, which have proven to be polymorphic for *P. cygnus*: Pcyg03, Pcyg04, Pcyg05, Pcyg11, Pcyg15, Pcyg18 (Kennington et al., 2010), and S28 (Groth, Lim, de Lestang, Beale, & Melville‐Smith, 2009). The 5′‐end of the forward primer from each locus was labeled with a fluorescent tag (FAM, NED, PET, VIC). PCRs were carried out in a 5‐μl volume with the following conditions: 1 μl of template DNA (10 ng), 1× Bioline MyTaq reaction buffer (containing 3 mmol/L MgCl_2_, 1 mmol/L of each dNTP, stabilizers and enhancers), 0.4 μmol/L of each primer, and 0.1 U/μl MyTaq DNA polymerase (Bioline). Amplifications were completed in an Eppendorf thermal cycler, after optimization of published annealing temperatures (Groth et al., 2009; Kennington et al., 2010). PCR products were analyzed on a 3700 Genetic Analyzer (Applied Biosystems, Inc), using an internal size standard (GS500 LIZ, ABI). Microsatellite alleles were identified by their sizes in base pairs using the software GENEMARKER v4.0 (SoftGenetics, State College, PA, USA). The genotypes of the males (*n* = 489) were sourced from a previous study (J. Loo et al., unpubl. data).

### 2.3 Data analysis

The program MICRO‐CHECKER (Van Oosterhout, Hutchinson, Wills, & Shipley, 2004) was used to detect genotyping or scoring errors, caused by null alleles, large allele dropout, or stutter peaks in the maternal genotypes. Duplicate samples were detected from the probability of genotype identity using GENALEX v. 6 (Peakall & Smouse, 2006). The probability of identity (*PI*, the average probability of different random individuals sharing the same genotype by chance) and a more conservative estimate of *PI*,* PIsibs*, which takes into account the presence of relatives, were also calculated using GENALEX. The same software was used to estimate the number of alleles and observed and expected heterozygosity for each locus from the maternal genotypes. Deviations from random mating were characterized using the *F*_IS_ statistic (inbreeding coefficient). Positive and negative *F*_IS_ values indicate a deficit or excess of heterozygotes relative to random mating, respectively. Linkage disequilibrium between each pair of loci was evaluated by testing the significance of association between genotypes. Inbreeding coefficient estimates were performed using FSTAT version 2.9.3 software package (Goudet, 2001). The program GENEPOP 3.1 (Raymond & Rousset, 1995) was used to assess conformity to Hardy‐Weinberg equilibrium (HWE). Probability values for deviation from HWE were estimated using the Markov chain method with 10,000 iterations.

Paternity was investigated by genotyping ~20 fertilized eggs obtained from each of the sampled females (see Table 2 for sample sizes). This level of sampling was based on analytical methods for calculating statistical power to detect multiple in highly fecund decapods (Veliz, Duchesne, Rojas‐Hernandez, & Pardo, 2017), although the number of females sampled in our study was below the recommended 50 females in that analysis (see Section 2.2). However, power analysis of sampling 20 eggs per female indicates that we had the ability to detect multiple spawning more than 99% of the time, if the contribution of sperm from two males was roughly equal. Even under the scenario of one male contributing the majority of sperm used to fertilize the egg mass (e.g., 90% of all sperm) our detection probability was still as high as 90%. Three different approaches were used to evaluate paternity: initial inference, the GERUD 2.0 software package (Jones, 2005), and the COLONY 2.0 software package (Wang, 2004; Wang & Santure, 2009). For the initial inference approach, paternal genotypes were inferred from nonmaternal alleles observed in the offspring. Multiple paternity was assumed only if more than two nonmaternal alleles occurred in more than one locus in the offspring, to allow for the possibility of mutation at one locus. We analyzed paternity with GERUD using it to reconstruct the minimum number of possible paternal genotypes. GERUD uses an exhaustive algorithm that takes into account information from patterns of Mendelian segregation and genotypic frequencies in the population. As GERUD does not accept missing data, the number of loci used in this study varied from 4 to 7. The parameter for the maximum number of fathers was set to four, and the runs were conducted with known maternal genotypes. Initial inference and GERUD assume that males are heterozygotes and that there is no allele sharing among fathers or between mother and father(s) and, consequently, they may be underestimating the number of fathers. Lastly, we used COLONY to assign parentage based on a maximum‐likelihood model. Unlike GERUD, this program accepts missing data. We used the default setting and all runs were performed with known maternal genotypes. Inferred paternal genotypes were compared to the genotypes of all sampled males. Multiple paternity was inferred for a clutch if at least two of the three methods (initial inference, GERUD, COLONY) detected more than one father.

In addition to the paternity analysis, inferred paternal genotypes for each clutch were compared to the genotype of the spermatophore attached to the corresponding mother. Genotype matching was carried out using the genotype identity option in GENALEX.

## 3 RESULTS

A total of 25 females carrying eggs (15 from Armstrong Bay and 10 from Kingston Reef) were genotyped. Based on genotype identity, one female from Kingston Reef was sampled twice. Of the remaining 24 females, 11 (~46%) had attached spermatophores with genotypes consisting of more than two alleles at a locus, indicating the presence of DNA from more than one male. (Note that we have previously confirmed that spermatophores consisting of multiple genotypes are unlikely to result from genotyping errors or contamination of female DNA and are therefore likely to result from multiple mating; J. Loo et al., unpubl. data.) A further five females had spermatophores with genotypes from a single male that did not match the genotype of the inferred sire, suggesting that these females had also mated with two or more males during the reproductive season. The maternal genotypes showed no evidence of null alleles, and there were no significant deviations from HWE at any locus (*p *>* *.05 in all cases). The probability of sampling identical maternal genotypes (*PI*) was 3.5 × 10^−7^, and a more conservative estimate of PI, which takes into account the presence of relatives, *PIsibs,* was 5.2 × 10^−3^. The number of alleles per locus ranged from 2 to 26, with observed heterozygosity ranging from 0.042 to 0.917 (Table 1).

**Table 1:** Genetic variation at microsatellite loci used in this study

Locus	*n*	*N*_a_	*H*_O_	*H*_E_	*F*_IS_
Pcyg03	24	5	0.250	0.323	0.25
Pcyg04	24	26	0.917	0.953	0.06
Pcyg05	24	7	0.708	0.710	0.02
Pcyg11	23	8	0.826	0.733	−0.11
Pcyg15	24	2	0.375	0.430	0.15
Pcyg18	24	2	0.042	0.041	0.00
S28	24	9	0.625	0.641	0.05
W25	22	9	0.591	0.543	−0.06

Estimates are based on maternal genotypes pooled across locations. *n*, sample size; *N*_a_, number of alleles; *H*_O_, observed heterozygosity; *H*_E_, expected heterozygosity; and *F*_IS_ inbreeding coefficient (*p* > .05 for all).

Based on initial inference, only one of 24 clutches showed multiple paternity. According to initial inference and GERUD, the minimum number of sires per clutch was one in 22 cases, with two cases of multiple paternity detected (minimum number of sires of two and three). The analysis in COLONY suggested three instances of multiple paternity (Table 2). Following a consensus approach, multiple paternity was identified only in the two clutches where at least two of the three methods used detected more than one sire. Interestingly, none of the 489 males that were sampled in this study was identified by COLONY as being a putative father of the 24 clutches examined.

**Table 2:** Minimum number of sires per clutch as estimated by initial inference, GERUD 2.0 and COLONY 2.0 runs with known maternal genotype

Clutch	No. of embryos analyzed	Initial inference	GERUD 2.0	COLONY 2.0	Spermatophore matched inferred parent	Multiple paternity
Kingston Reef						
1	20	1	1	1	Yes	No
2	20	1	1	1	POLY	No
3	40	1	1	1	No	No
4	20	1	1	1	Yes	No
5	19	1	1	1	Yes	No
6	18	1	1	2	POLY	No
7	19	1	1	1	POLY	No
8	20	3	3	3	No	Yes
9	20	1	1	1	POLY	No
Armstrong Bay						
1	18	1	1	1	Yes	No
2	20	1	1	1	Yes	No
3	19	1	1	1	POLY	No
4	19	1	1	1	No	No
5	19	1	1	1	POLY	No
6	20	1	1	1	Yes	No
7	20	1	2	2	POLY	Yes
8	18	1	1	1	No	No
9	19	1	1	1	POLY	No
10	20	1	1	1	Yes	No
11	20	1	1	1	Yes	No
12	19	1	1	1	No	No
13	20	1	1	1	POLY	No
14	20	1	1	1	POLY	No
15	20	1	1	1	POLY	No

POLY indicates cases of polyandry where the spermatophore consisted of more than one genotype (i.e., three or more alleles at least one locus). Criteria to determine multiple paternity: detection of a minimum of two sires per clutch by at least two of the three methods.

Inferred paternal genotypes (from fertilized eggs) were compared with the genotypes of the spermatophores collected from the corresponding egg‐carrying females (Table 2). Of these, eight (33%) matched the genotype of the spermatophore attached to the mother. The remaining inferred paternal genotypes did not match the genotype of the spermatophore attached to the mother (17%) or could not be compared to the genotype of the spermatophore attached to the mother because the spermatophore contained ejaculates from more than one male.

## 4 DISCUSSION

Our study confirms that while multiple mating by female *P. cygnus* is relatively common, incidences of multiple paternity are extremely rare. We found that spermatophores attached to females often came from two or more males, confirming our previous evidence that polyandry is widespread in natural populations of *P. cygnus* (J. Loo et al., unpubl. data). Despite this evidence for female multiple mating, however, we found limited evidence of multiple paternity.

One simple explanation for the disparity between patterns of female multiple mating and the incidence of multiple paternity is that our sampling protocol may have resulted in low statistical power. Recently, Veliz et al. (2017) developed an analytical method that assessed the statistical power to detect multiple paternity in crabs. According to their analysis, sampling 20 eggs from *n *=* *50 females yields very high statistical power to detect multiple paternity, even in highly fecund species with 1 × 10^6^ eggs per clutch. In our study, we were restricted to approx. half this number of females, possibly restricting our ability to fully detect cases of multiple paternity. However, Veliz et al. (2017) also found that studies employing reduced levels of sampling (in terms of clutch size and number of females sampled) also had high power (~98%) to detect multiple paternity. In the present study, we suspect that even if we had improved our statistical power with greater levels of sampling, based on our power analysis, cases of multiple paternity would still have been rare and/or paternity would have been heavily skewed toward a single male in most cases.

A second possible explanation for the disparity between patterns of female multiple mating and the incidence of multiple paternity is that females mate consecutively with individual males each time they produce a batch of eggs, and that our observed patterns of (largely single) paternity reflect a pattern of serial monogamy over the course of the breeding season. As we note above, in *P. cygnus* mating entails the attachment of the male's spermatophore (tar spot) to the underside of the female, which is partially eroded by the female during fertilization and is only sloughed off in the following molting. Subsequent matings within the same reproductive season (molt cycle) involve a male depositing a fresh spermatophoric mass on top of the previously eroded (used) spermatophore (de Lestang & Melville‐Smith, 2006). This can lead to the spermatophoric mass on a female being dominated by a single sire (by virtue of their positioning and numerical supremacy) while still containing the DNA from multiple sires. This is reflected by the high incidence of multiple mating and low occurrence of multiple paternity. However, when double spawning has been observed, it is more likely to occur in the larger females (Chittleborough, 1976; Chubb, 1991; de Lestang & Melville‐Smith, 2006). This pattern of larger females spawning twice in a season has also been observed in other spiny lobsters (Briones‐Fourzán & Lozano‐Alvarez, 1992; Gomez, Junio, & Bermas, 1994; Macfarlane & Moore, 1986). While these observations support the idea of serial monogamous matings, we have confirmed elsewhere that females carrying spermatophores from more than one male had a wide range of body sizes (carapace length 69.5–106.5 mm) and there was no evidence of higher rates of multiple mating in larger females (J. Loo et al., unpubl. data).

A final explanation for the high levels of polyandry observed in this study is that females mate with multiple males between fertilization events and sperm competition and/or female cryptic choice function to refine fertilization success in favor of a subset of mated males. This explanation also accounts for the disparity between patterns of multiple mating (high incidence) and multiple paternity (low incidence). In species where females store spermatophores externally, sperm competition can occur when a male displaces or removes the spermatophore from the female. For example, in the freshwater crayfish *Austropotamobius italicus*, males remove and consume all (or most) of the spermatophores from previously mated males before releasing their own sperm (Galeotti et al., 2007). The occurrence of cryptic female choice is often more difficult to infer due to the diversity of possible underlying mechanisms and their interactions with sperm competition (reviewed by Firman, Gasparini, Manier, & Pizzari, 2017). Consequently, evidence of cryptic female choice in decapods is limited, but compelling examples of the phenomenon come from studies of other marine species with external sperm deposition (e.g., Japanese pygmy squid, *Idiosepius paradoxus*; Sato, Yoshida, & Kasugai, 2017) and external fertilization (ocellated wrasse, *Symphodus ocellatus*; Alonzo, Stiver, & Marsh‐Rollo, 2016). Cryptic female choice has been proposed in decapods based on behavioral observations, including failed copulations (Bauer, 1996; Diesel, 1990; Ra'anan & Sagi, 1985) and delayed oviposition (Thiel & Hinojosa, 2003). However, such observations do not demonstrate cryptic female choice by themselves. More direct evidence of cryptic female choice in decapods comes from observations of females removing or displacing spermatophores. For example, removal of sperm has been reported for rock shrimps, *R. typus* (Thiel & Hinojosa, 2003) and anecdotally in the spiny lobster *Panulirus guttatus* (Magallon‐Gayon, Briones‐Fourzan, & Lozano‐Alvarez, 2011). In *P. cygnus*, spermatophores are attached externally to the female and fertilization is temporally decoupled from mating, suggesting that there is some opportunity for cryptic female choice in this system. We clearly require further observational and/or experimental studies to identify the mechanisms that generate paternity biases in this system.

In summary, this study revealed limited evidence of multiple paternity in *P. cygnus*, despite the high frequency of multiple mating. This suggests that although females mated with more than one male, fertilization was attained by only a subset (one or two) of these males. We have yet to determine whether female multiple mating is adaptive (e.g., because it enables females to ensure that sperm from intrinsically “good” males win the race to fertilize their eggs; Curtsinger, 1991; Yasui, 1997) or is a by‐product of accumulated matings that take place throughout the breeding season. However, our observations of high levels of female multiple mating reveal the potential for postmating sexual selection to operate in this system. We eagerly await follow‐up studies designed to elucidate such mechanisms and test for possible reproductive benefits of polyandry.

## AUTHOR CONTRIBUTIONS

JL, WK, JE, JH, and SL conceptualized and planned the study; JL, JH, and SL were responsible for field collections; JL conducted molecular work and carried paternity analyses; JL and WK were principally responsible for statistical analyses, with input from all authors; all authors were involved in writing and editing the manuscript.

## CONFLICT OF INTEREST

The authors declare no conflicts of interest.
